# The Association between Glomerular Filtration Rate Estimated Using Different Equations and Mortality in the Japanese Community-Based Population: The Yamagata (Takahata) Study

**DOI:** 10.1155/2018/9191832

**Published:** 2018-02-19

**Authors:** Asami Kabasawa, Tsuneo Konta, Natsuko Suzuki, Keita Kamei, Sayumi Watanabe, Akira Araumi, Eri Matsuki, Soichiro Kon, Midori Oyama, Kazunobu Ichikawa, Kenichi Ishizawa, Yoshiyuki Ueno, Hidetoshi Yamashita, Takamasa Kayama, Isao Kubota

**Affiliations:** ^1^Department of Cardiology, Pulmonology, and Nephrology, Yamagata University School of Medicine, Yamagata, Japan; ^2^Department of Public Health and Hygiene, Yamagata University Graduate School of Medical Science, Yamagata, Japan; ^3^Global Center of Excellence, Yamagata University School of Medicine, Yamagata, Japan

## Abstract

**Background:**

To evaluate renal function, the indices of estimated glomerular filtration rate (eGFR) obtained using several equations, including the Japanese versions of the serum creatinine-based MDRD equation (eGFRcreat), Chronic Kidney Disease Epidemiology Collaboration equation (eGFR-EPI), and serum cystatin C-based equation (eGFRcys), are utilized. This study prospectively examined the association between these eGFR values and all-cause mortality during a 12-year observational period in a community-based population.

**Methods and Results:**

The subjects of this study were 1312 participants undergoing a health checkup, aged ≥40 years. In the total population, the mean eGFR values (mL·min^−1^·1.73 m^−2^) were 81.5 for eGFRcreat, 78.1 for eGFR-EPI, and 76.6 for eGFRcys. There were 141 deaths during the observation period, and the area under the receiver operating characteristic curve for predicting mortality was 0.59 for eGFRcreat, 0.67 for eGFR-EPI, and 0.70 for eGFRcys (all *P* < 0.01). In the Cox proportional analysis adjusted for age and sex, eGFRcys, but not eGFRcreat and eGFR-EPI, showed a significant association with all-cause mortality (per 15 mL·min^−1^·1.73 m^−2^ decrease: hazard ratio 1.40, 95% confidence interval 1.18–1.67).

**Conclusions:**

This study revealed that eGFRcys showed lower values than eGFRcreat and eGFR-EPI and was significantly associated with all-cause mortality in the Japanese community-based population.

## 1. Background

The association between renal dysfunction, cardiovascular diseases (CVDs), and total mortality in the general population has been reported previously [1]. The same finding is observed worldwide, including in Japan [2, 3]. Therefore, from an epidemiological point of view, renal function, which is usually expressed as the value of the estimated glomerular filtration rate (eGFR), is used to predict events including not only end-stage kidney disease but also CVD and premature death.

In the calculation of eGFR, several equations including the serum creatinine-based Modification of Diet in Renal Disease (MDRD) equation, Chronic Kidney Disease Epidemiology Collaboration (CKD-EPI) equation, and serum cystatin C-based equation are utilized. The MDRD equation was developed in 1999, using the values of age, sex, and serum creatinine level [4]. Thereafter, to overcome the defect of the MDRD equation of overestimating renal function in subjects with normal or mildly reduced renal function (e.g., GFR ≥ 60 mL·min^−1^·1.73 m^−2^), the CKD-EPI formula was developed in 2009 [5]. Additionally, the serum cystatin C-based equation, which uses age, sex, and serum cystatin C level, was developed in 2012 [6]. Because the serum cystatin C level is less affected by muscle mass, diet, and exercise than the serum creatinine level, the serum cystatin C-based equation is more suitable for cases in which muscle mass is reduced because of limb amputation, a lean body, and long-term bedridden state or for cases of high muscle mass [7].

However, these formulas were developed for the Western population and showed a nonnegligible discrepancy between the eGFR and the actually measured GFR in Japanese subjects. Therefore, for the Japanese population, eGFR values calculated using the Japanese versions of the MDRD equation (eGFRcreat) [8], CKD-EPI equation (eGFR-EPI) [9], and serum cystatin C-based equation (eGFRcys) [10] were established.

In recent years, it has been reported that the CKD-EPI equation is superior to the MDRD equation in predicting the development of CVD [11] and that the use of the serum cystatin C level alone or in combination with the creatinine level strengthens the association between eGFR and end-stage kidney disease and mortality [12]. Moreover, a Japanese study in the Iwate region showed that the CKD-EPI equation is more useful for predicting CVD than the Japanese MDRD formula [13]. Although these studies showed a difference in the association between the two equations, it is not clear which marker (eGFRcreat, eGFR-EPI, or eGFRcys) is most useful for predicting death in the Japanese community-based population. To clarify this issue, a prospective cohort study was conducted in the Japanese community-based population.

## 2. Methods

The Yamagata (Takahata) study is a community-based cohort study that recruited participants undergoing an annual health checkup in Takahata town. In this study, 1312 subjects who underwent medical examination including testing for the serum concentration of creatinine and cystatin C between 2004 and 2006 agreed to participate and were followed up for 12 years (median, 11.3 years). Details on the research design, recruitment procedures, and population profiles are described elsewhere [14]. The institutional ethics committees of Yamagata University School of Medicine and the town of Takahata approved this study (Yamagata University, April 2006, no. 1), and all subjects provided written informed consent. The procedures were performed in accordance with the Helsinki Declaration.

To investigate the association between renal function and prognosis, a follow-up survey was performed annually until the end of 2015. The causes of death were determined by reviewing death certificates through the end of 2015. The death code (International Classification of Diseases, 10th revision) and the date and place of death were reviewed [14].

At baseline, medical history and clinical symptoms were investigated using a self-answered questionnaire. Systolic blood pressure and diastolic blood pressure were measured in the sitting position after the subject had rested for 5 min or longer, using a mercury pressure gauge. Hypertension was defined as a systolic blood pressure of ≥140 mmHg or a diastolic blood pressure of ≥90 mmHg or the use of oral antihypertensive drugs. Obesity was defined as a body mass index of ≥25.0 kg/m^2^. Diabetes was defined as a fasting blood glucose level of ≥126 mg/dL and hemoglobin A1c of ≥6.5% or the use of a hypoglycemic drug. Hyperlipidemia was defined as a serum total cholesterol level of ≥220 mg/dL or the use of oral therapeutic agents for hyperlipidemia.

Serum creatinine level was measured using the enzymatic method. Serum cystatin C level was measured using latex agglutination turbidimetry. eGFRs according to the serum creatinine level (eGFRcreat) and serum cystatin C level (eGFRcys) were obtained using the Japanese GFR calculation formulas prepared by the Japanese Society of Nephrology [8, 10]. eGFR-EPI was calculated using the CKD-EPI equation with coefficients for the Japanese population [9].

eGFRcreat:

(1) $$\begin{array}{c} Men\text{: }eGFRcreat\;\left(mL\cdot \min^{-1}\cdot 1.73\;m^{-2}\right)=194\times {Cr}^{-1.094}\times {age}^{-0.287},\\ Women\text{: }eGFRcreat\;\left(mL\cdot \min^{-1}\cdot 1.73\;m^{-2}\right)=194\times {Cr}^{-1.094}\times {age}^{-0.287}\times 0.739. \end{array}$$

eGFR-EPI:

(2) $$\begin{array}{c} Men\text{: }eGFR‐EPI\;\left(mL\cdot \min^{-1}\cdot 1.73\;m^{-2}\right)=141\times {\left(\frac{Cr}{\kappa }\right)}^{\alpha }\times 0.993^{age}\times 0.813,\\ Women\text{: }eGFR‐EPI\;\left(mL\cdot \min^{-1}\cdot 1.73\;m^{-2}\right)=141\times {\left(\frac{Cr}{\kappa }\right)}^{\alpha }\times 0.993^{age}\times 1.018\times 0.813\;\left(\kappa :men\;0.9,women\;0.7\right),\left(\alpha :Cr>\kappa ,\alpha =-1.209,Cr\leq \kappa ,men-0.411,women-0.329\right). \end{array}$$

eGFRcys:

(3) $$\begin{array}{c} Men\text{: }eGFRcys\;\left(mL\cdot \min^{-1}\cdot 1.73\;m^{-2}\right)=\left(104\times Cys‐C\times 0.996^{age}\right)-8,\\ Women\text{: }eGFRcys\;\left(mL\cdot \min^{-1}\cdot 1.73\;m^{-2}\right)=\left(104\times Cys‐C^{-1.019}\times 0.996^{age}\times 0.929\right)-8. \end{array}$$

### 2.1. Statistical Analysis

Data are expressed as mean ± standard deviation, unless otherwise indicated. Both unadjusted and age- and sex-adjusted Cox-proportional hazard model analyses were performed to examine the relationship between eGFRcreat, eGFR-EPI, and eGFRcys and all-cause death. All statistical analyses were performed using JMP version 10 (SAS Institute Inc., Cary, NC, USA). The area under a receiver operating characteristic (ROC) curve (AUC) for all-cause mortality was evaluated. To compare the AUC by using eGFR, eGFRcys, and eGFR-EPI, the DeLong test in the pROC version 1.10.0 package of R statistics (http://www.r-project.org) was used. A significant difference was defined as *P* < 0.05.

## 3. Results

The baseline characters of all 1312 subjects (588 men, 724 women) are shown in Table 1. The mean age was 63.9 years. The mean values of serum creatinine and serum cystatin C levels were 0.67 ± 0.16 mg/dL and 0.95 ± 0.19 mg/L, respectively. The mean values of eGFR-EPI and eGFRcys were lower than that of eGFRcreat (81.5 ± 17.0 mL·min^−1^·1.73 m^−2^ for eGFRcreat, 78.1 ± 11.2 mL·min^−1^·1.73 m^−2^ for eGFR-EPI, and 76.6 ± 16.4 mL·min^−1^·1.73 m^−2^ for eGFRcys). During the 12-year follow-up period, 141 all-cause deaths occurred.

**Figure 1:**
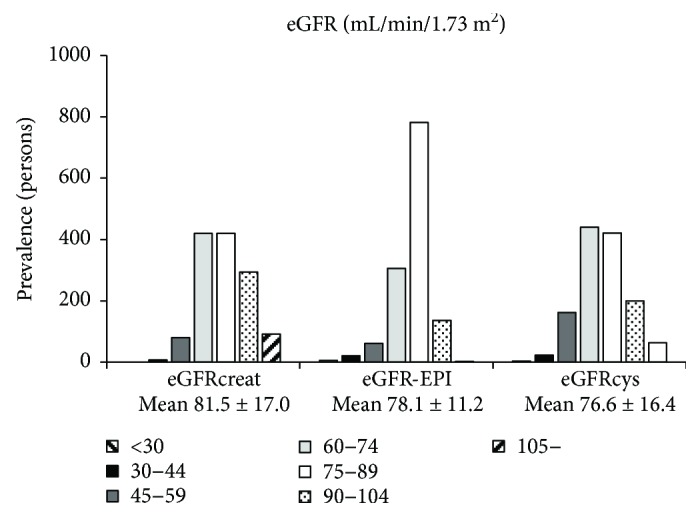
Distribution of eGFRcreat, eGFR-EPI, and eGFRcys. The proportion of subjects with renal insufficiency (eGFR < 60 mL·min^−1^·1.73 m^−2^) was 6.6% for eGFRcreat, 6.7% for eGFR-EPI, and 14.3% for eGFRcys. eGFR: estimated glomerular filtration rate.

**Figure 2:**
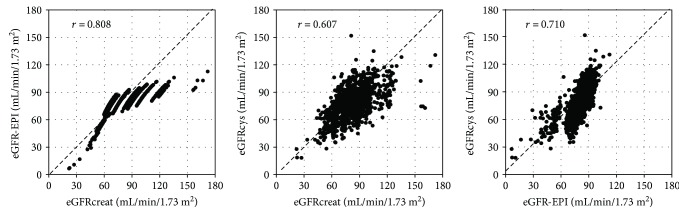
Correlation between eGFRcreat, eGFR-EPI, and eGFRcys. The correlation between these eGFR values was significantly positive.

First, we investigated the distribution and correlation of these eGFR values. The proportions of subjects with CKD stage 3A (eGFR 45–59 mL·min^−1^·1.73 m^−2^), 3B (eGFR 30–44 mL·min^−1^·1.73 m^−2^), and 4-5 (eGFR < 30 mL·min^−1^·1.73 m^−2^) were 6.1%, 0.3%, and 0.2%, respectively, for eGFRcreat; 4.6%, 1.6%, and 0.5%, respectively, for eGFR-EPI; and 12.2%, 1.8%, and 0.3%, respectively for eGFRcys (Figure 1). The proportion of subjects with renal insufficiency (eGFR < 60 mL·min^−1^·1.73 m^−2^) was 6.6% for eGFRcreat, 6.7% for eGFR-EPI, and 14.3% for eGFRcys. The correlation between the eGFR values estimated using these equations was significantly positive, and the correlation coefficient was 0.808 between eGFRcreat and eGFR-EPI, 0.607 between eGFRcreat and eGFRcys, and 0.710 for eGFR-EPI and eGFRcys (Figure 2).

**Figure 3:**
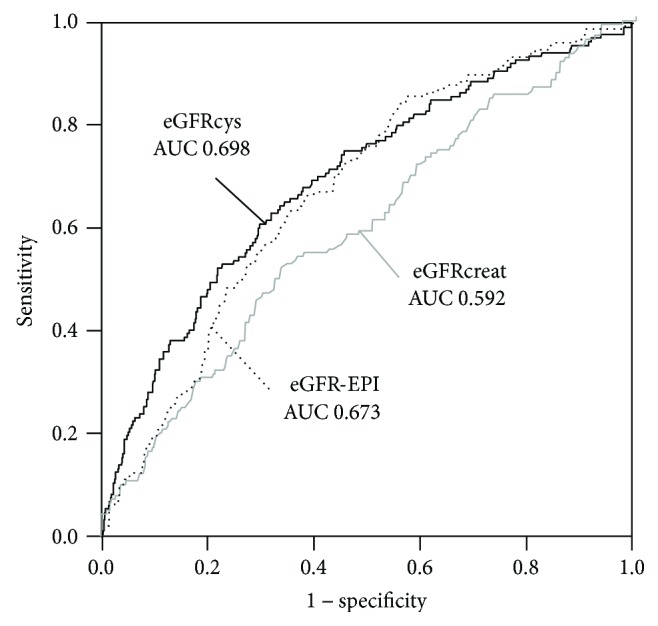
ROC curve for predicting all-cause mortality by eGFRcreat, eGFR-EPI, and eGFRcys. A significant difference was detected between eGFRcreat and eGFR-EPI and between eGFRcreat and eGFRcys but not between eGFR-EPI and eGFRcys. AUC: area under the curve.

Next, we examined the association between these eGFR values and mortality. The AUC for predicting all-cause mortality was 0.59 for eGFRcreat, 0.67 for eGFR-EPI, and 0.70 for eGFRcys (all *P* < 0.01) (Figure 3). In the DeLong test to compare the AUC, a significant difference was detected between eGFRcreat and eGFR-EPI (*P* = 0.02) and between eGFRcreat and eGFRcys (*P* < 0.01) but not between eGFR-EPI and eGFRcys (*P* = 0.679).

**Figure 4:**
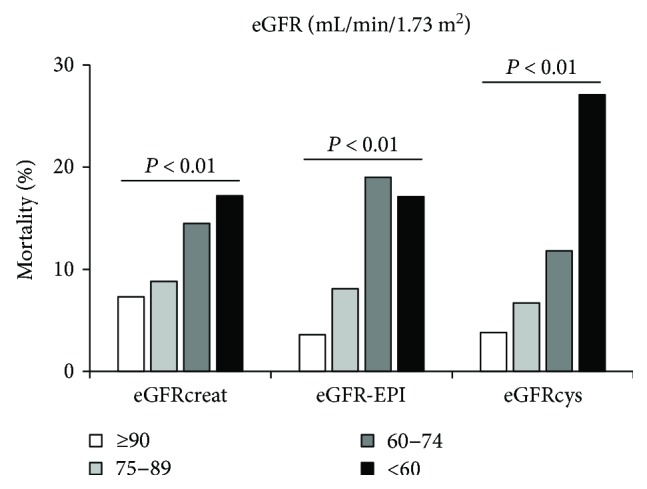
All-cause mortality by baseline eGFR levels. Along with the decrease in baseline eGFR values, the mortality rate was significantly increased. eGFR: estimated glomerular filtration rate.

Further, we examined the risk for 12-year mortality in subjects with reduced renal function. Along with the decrease in baseline eGFR values, the mortality rate was significantly increased. The mortality rate (%) when eGFR ≥ 90, 75–89, 60–74, and <60 mL·min^−1^·1.73 m^−2^ was 7.3, 8.8, 14.5, and 17.2, respectively, for eGFRcreat; 3.6, 8.1, 19.0, and 17.1, respectively, for eGFR-EPI; and 3.8, 6.7, 11.8, and 27.1, respectively, for eGFRcys (Figure 4). The number of the subjects with eGFRcreat > 60 and eGFRcys < 60 was 139 (10.9% of total population), and among them, there were 37 all-cause deaths (mortality rate 26.6).

In Cox proportional hazard analysis, the unadjusted risk for all-cause mortality was significantly increased with the decrease in eGFRcreat, eGFR-EPI, and eGFRcys. However, after the adjustment for age and sex, a significant association was observed for eGFRcys but not for eGFRcreat and eGFR-EPI. The hazard ratio (HR) (95% confidence interval (CI)) of eGFR 75–89, 60–74, and <60 mL·min^−1^·1.73 m^−2^ with eGFR ≥ 90 mL·min^−1^·1.73 m^−2^ as the reference was 1.04 (0.66–1.66), 1.37 (0.90–2.12), and 1.23 (0.66–2.22), respectively, for eGFRcreat; 0.82 (0.37–2.20), 1.07 (0.44–3.04), and 0.99 (0.37–2.98), respectively, for eGFR-EPI; and 0.94 (0.50–1.83), 1.28 (0.70–2.49), and 2.52 (1.33–5.08), respectively, for eGFRcys (Table 2). Furthermore, we examined the age- and sex-adjusted mortality risk per 15 mL·min^−1^·1.73 m^−2^ decrease of baseline eGFR. We found that the increase in mortality risk was significant for eGFRcys (HR 1.40, 95%CI 1.18–1.67) but not for eGFRcreat (HR 1.10, 95%CI 0.95–1.28) and eGFR-EPI (HR 1.15, 95 CI% 0.94–1.38) (Table 2). The analysis using the mean values of eGFRcreat and eGFRcys showed that the AUC for predicting all-cause mortality was 0.66 (*P* < 0.01) and that the age- and sex-adjusted mortality risk per 15 mL·min^−1^·1.73 m^−2^ decrease of baseline eGFR was significant (HR 1.26, 95%CI 1.06–1.51), indicating that the predictive ability of the mean values of eGFRcreat and eGFRcys was not better than eGFRcys alone.

**Figure 5:**
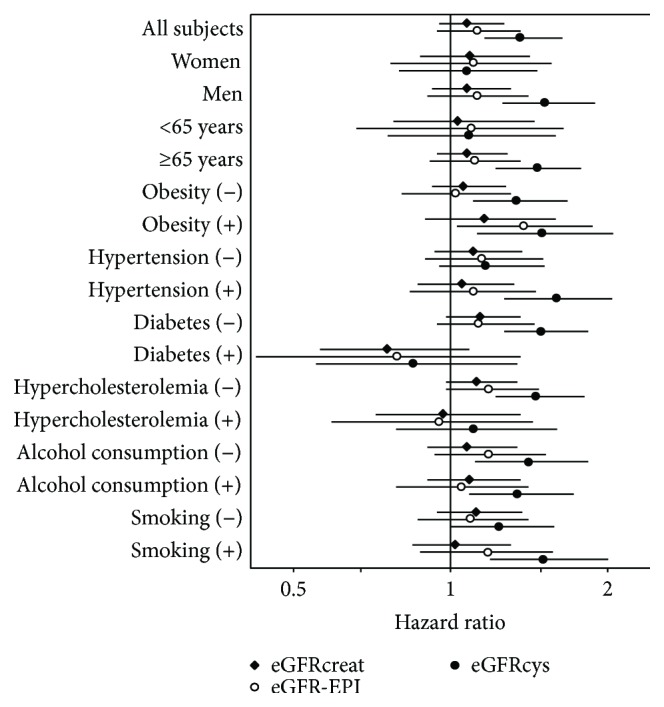
Hazard ratio for all-cause mortality of baseline eGFR levels (per 15 mL/min/1.73m^2^ decrease). The hazard ratios of the per 15 mL·min^−1^·1.73 m^−2^ decreases of baseline eGFR were higher for eGFRcys than for eGFRcreat and eGFR-EPI in most subgroups.

In addition, we performed subgroup analyses in men/women, subjects aged <65 years/≥65 years, and subjects with/without obesity, hypertension, diabetes, hypercholesterolemia, alcohol consumption, and smoking. These analyses showed a similar finding: the HRs of the per-15 mL·min^−1^·1.73 m^−2^ decreases of baseline eGFR were higher for eGFRcys than for eGFRcreat and eGFR-EPI in most subgroups (Figure 5).

## 4. Discussion

In this study, we showed for the first time that the values of eGFRcys were lower than those of eGFR-EPI and eGFRcreat and that a significant association with mortality was observed for eGFRcys but not for eGFRcreat and eGFR-EPI in the Japanese community-based population.

Previous studies have examined the accuracy of various equations for estimating GFR. In Japanese subjects, it has been reported that the median bias (difference between eGFR and measured GFR) of eGFRcreat, eGFR-EPI, and eGFRcys was 1.9, 0.8, and 0.3, respectively, and their 30% accuracy was 75%, 75%, and 78%, respectively [15]. This indicates that the performance of the eGFR equations is not significantly different among each other. In this study, the average value of eGFRcys was lower than those of eGFR-EPI and eGFRcreat. One of the possible reasons for this discrepancy might be that most of the participants undergoing health checkups have preserved renal function and are elderly. It is reported that eGFRcys shows better performance than eGFRcreat in subjects with normal and mildly reduced GFR [16] and in subjects with reduced muscle mass [7]. Additionally, it is documented that eGFRcreat tends to be underestimated in young persons and overestimated in middle-aged and elderly subjects with GFR ≥ 60 mL·min^−1^·1.73 m^−2^ [13]. However, as we did not measure the true GFR values using inulin levels, we cannot determine which eGFR value is the most accurate in this study.

Concerning the association between eGFR and prognosis, some studies have examined eGFRcreat and eGFR-EPI. Matsushita et al. reported that the categorization by eGFR-EPI was more correlated with prognosis than that by eGFRcreat [11]. Ohsawa et al. showed that eGFR-EPI more accurately reflects renal function and is more correlated with death than eGFRcreat [13]. Other studies reported that the association of eGFRcys with mortality was stronger than that of eGFRcreat in subjects with type 2 diabetes [17], the elderly [18], and the Western population [12]. Our study further clarified that the association with mortality was stronger for eGFRcys than for eGFRcreat and eGFR-EPI in the Japanese community-based population.

The findings of this study have important clinical implications. eGFRcreat is mainly used in clinical practice; however, this study suggested that a substantial number of subjects with borderline renal insufficiency, showing eGFRcys < 60 mL·min^−1^·1.73 m^−2^, might be overlooked in health checkups. Furthermore, the association with all-cause mortality was stronger for eGFRcys than for eGFRcreat and eGFR-EPI. Although convenience of use and cost-effectiveness are other important issues to consider, eGFRcys may be a better marker for evaluating the risk for premature death in participants of health checkups.

This study has several notable limitations. First, we did not perform the gold standard method for GFR measurement (inulin clearance method); therefore, we do not know the difference between the actual GFR values and the eGFR values obtained with each equation. Second, the number of events was not enough to perform an analysis according to the cause of deaths, and a multivariate analysis adjusted for other confounders such as diabetes mellitus and smoking. Third, the number of subjects with eGFR < 30 mL·min^−1^·1.73 m^−2^ was small in this study; therefore, it is unknown whether our findings can be applied to subjects with severe renal dysfunction. Fourth, there is a possibility that the small number of subjects with eGFR < 60 in eGFR-EPI and eGFRcreat causes an underestimation of the association between these eGFR values and all-cause mortality in this study.

In conclusion, this study revealed that eGFRcys showed lower values than eGFRcreat and eGFR-EPI and that the association with mortality was stronger for eGFRcys than for eGFRcreat and eGFR-EPI in the Japanese community-based population, suggesting that eGFRcys might be a better marker for health checkups.

## Figures and Tables

**Table 1:** Baseline characteristics of the study subjects.

	Total	Males	Females
Number	1312	588	724
Age (years)	63.9 ± 9.9	64.6 ± 9.8	63.6 ± 9.9
Smoker (%)	30.6	59.0	7.6
Alcohol consumption (%)	40.7	72.8	14.6
Obesity (%)	31.6	32.0	31.2
Hypertension (%)	43.1	48.6	38.5
Diabetes (%)	5.6	6.9	4.4
Hypercholesterolemia (%)	31.1	22.1	38.4
Systolic blood pressure (mmHg)	134.2 ± 15.7	136.5 ± 15.5	132.4 ± 15.6
Diastolic blood pressure (mmHg)	78.7 ± 9.9	81.5 ± 9.8	76.5 ± 9.4
Serum total cholesterol (mg/dL)	199.6 ± 30.7	192.0 ± 29.7	205.8 ± 30.1
Fasting blood sugar (mg/dL)	94.2 ± 15.1	96.5 ± 16.9	92.3 ± 13.2
HbA1c (%)	5.64 ± 0.64	5.63 ± 0.68	5.66 ± 0.60
Serum creatinine (mg/dL)	0.67 ± 0.16	0.77 ± 0.16	0.59 ± 0.11
Serum cystatin C (mg/L)	0.95 ± 0.19	0.99 ± 0.20	0.92 ± 0.18
eGFRcreat (mL/min/1.73 m^2^)	81.5 ± 17.0	81.5 ± 16.6	81.4 ± 17.3
eGFR-EPI (mL/min/1.73 m^2^)	78.1 ± 11.2	77.1 ± 11.9	78.9 ± 10.5
eGFRcys (mL/min/1.73 m^2^)	76.6 ± 16.4	75.8 ± 15.9	77.2 ± 16.7

eGFR: estimated glomerular filtration rate. Parametric variables are expressed as mean ± SD.

**Table 2:** Hazard ratios for mortality by the baseline eGFR.

	eGFRcreat		eGFR-EPI		eGFRcys	
eGFR category						
Unadjusted	HR (95%CI)	*P* value	HR (95%CI)	*P* value	HR (95%CI)	*P* value
eGFR ≥ 90	Reference		Reference		Reference	
75–89	1.21 (0.76–1.93)	0.416	2.17 (1.03–5.58)	0.042	1.38 (0.75–2.68)	0.307
60–74	2.17 (1.45–3.34)	<0.001	5.39 (2.54–13.9)	<0.001	2.59 (1.49–4.84)	<0.001
<60	2.47 (1.34–4.38)	0.005	4.64 (19.3–12.8)	<0.001	6.63 (3.82–12.4)	<0.001
Age, gender-adjusted	HR (95%CI)	*P* value	HR (95%CI)	*P* value	HR (95%CI)	*P* value
eGFR ≥ 90	Reference		Reference		Reference	
75–89	1.04 (0.66–1.66)	0.808	0.82 (0.37–2.98)	0.67	0.94 (0.50–1.83)	0.840
60–74	1.37 (0.90–2.12)	0.143	1.07 (0.44–3.04)	0.883	1.28 (0.70–2.49)	0.432
<60	1.23 (0.66–2.22)	0.511	0.99 (0.37–2.98)	0.982	2.52 (1.33–5.08)	0.004
eGFR values						
Per-15 mL/min/1.73 m^2^ decrease	HR (95%CI)	*P* value	HR (95%CI)	*P* value	HR (95%CI)	*P* value
Unadjusted	1.35 (1.17–1.56)	<0.001	1.55 (1.34–1.78)	<0.001	1.79 (1.55–2.07)	<0.001
Age, gender-adjusted	1.10 (0.95–1.28)	0.216	1.15 (0.94–1.38)	0.177	1.40 (1.18–1.67)	<0.001

eGFR: estimated glomerular filtration rate; HR: hazard ratio; CI: confidence interval.

## Data Availability

Upon a request, data is available from the corresponding author.
